# Supplementary material for: Material-Sparing Feasibility Screening for Hot Melt Extrusion
Source: Pharmaceutics. 2024 Jan 5;16(1):76. doi: 10.3390/pharmaceutics16010076 (PMC10819182; doi:10.3390/pharmaceutics16010076)
Supplement: Supplementary file 1 [file pharmaceutics-16-00076-s001.zip › pharmaceutics-2755136-supplementary.pdf]

Supplemental Information

Degradation pre-screening

Selection of parameters

15 minutes was selected as the hold time for the degradation pre-screening studies. Because no mixing is employed, it was assumed that times longer than the HME mixing time would be needed. 15 minutes was selected as it represents the high-end of typical residence time distributions (measured in-house) experienced in an extruder.

A 50:50 mixture is used, even if 50wt% is not the desired drug loading, as it gives the best balance of signal from both components and makes interpretation straightforward. If neither the API or polymer's degradation is impacted by the presence of the other, than the 50/50 mixture should appear in the middle of the pure components: at a given temperature the weight loss should be the average of the loss determined for the polymer and the API for TGA, and the color should be an 'average hue' between what is observed on the API and polymer during visual testing. If this is not the case, it is obviously so – more than relying on lower loadings of API that might be more representative of the desired ASD composition. The idea is to understand if the degradation temperature is impacted by the materials' presence with one another.

Initial testing was run in 10°C increment, as demonstrated in Figure S1 for the visual assessment of RTV and PXCM. It was decided to run the tests in 20°C increments to minimize experimental iteration and time (especially for the visual testing), while still having enough granularity in the temperatures. Similar studies were done with the TGA method.

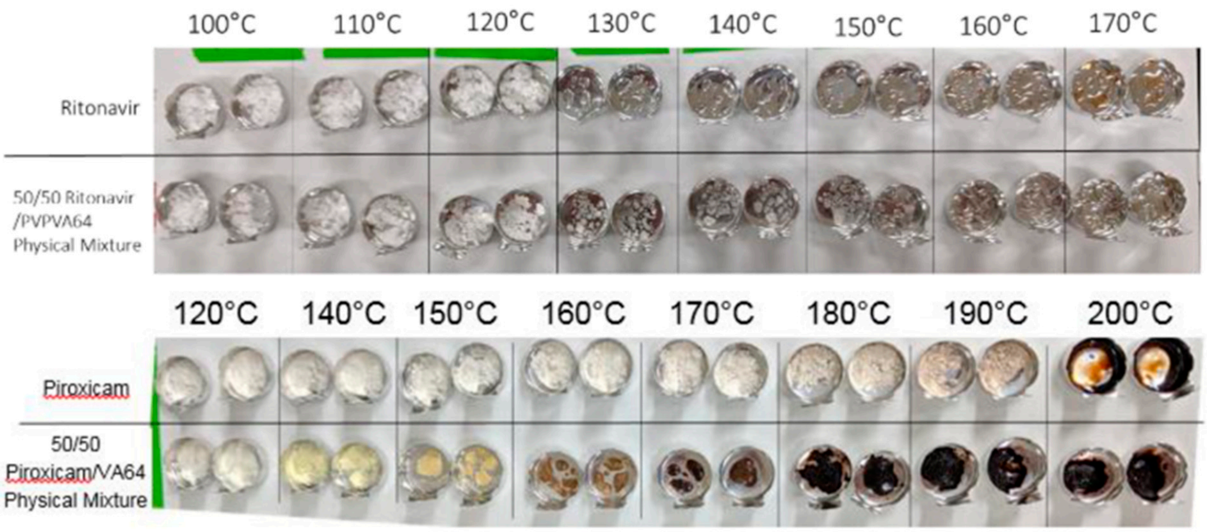

Figure S1. Results from the visual analysis with 10°C increments between samples.

For the TGA testing specifically, despite 0.5% loss potentially being considered significant degradation the 0.5-1.0 wt% mass loss thresholds were chosen since volatile loss is not always due to degradation and the testing is run in tandem with the visual results. Since neither of these tests are API-specific, the degradation-pre screening shouldn't be overly limiting since the final recommendation will ultimately be based on the HPLC results of the MiniMixer processed samples.

Additional analytical techniques explored

FTIR, Raman, and ^{1}H-NMR were also evaluated as possible techniques for degradation screening, but ultimately eliminated as candidates. FTIR and Raman spectroscopy did not have significant sensitivity to detect low level degradation. They were further convoluted by the changes in peak intensity and broadness between the amorphous and crystalline states of the API. ^{1}H-NMR also had sensitivity issues, along with some other challenges. It was expected that new peaks would appear when degradants were present, but the

polymer peaks have the possibility of interfering with observable API degradant peaks. Additionally, peak shifting can occur, that is not necessarily indicative of degradation. For example, when evaluating PXCM with a different polymer (Eudragit EPO), the PXCM peaks were shifted because the polymer had deprotonated the API and not because of degradation.

PXRD quantitation

To estimate the amount of solubilized API (i.e. the amorphous fraction), the residual crystalline peaks identified by PXRD were used for quantitation. First, diffractograms were fitted using the SmartLab Studio II software, ensuring consistent background fitting and minimized residuals. Figure S3 shows the representative fitted diffractograms compared to the experimental traces for the PXCM samples, and Figure S4 shows the identified peaks along with the residual trace.

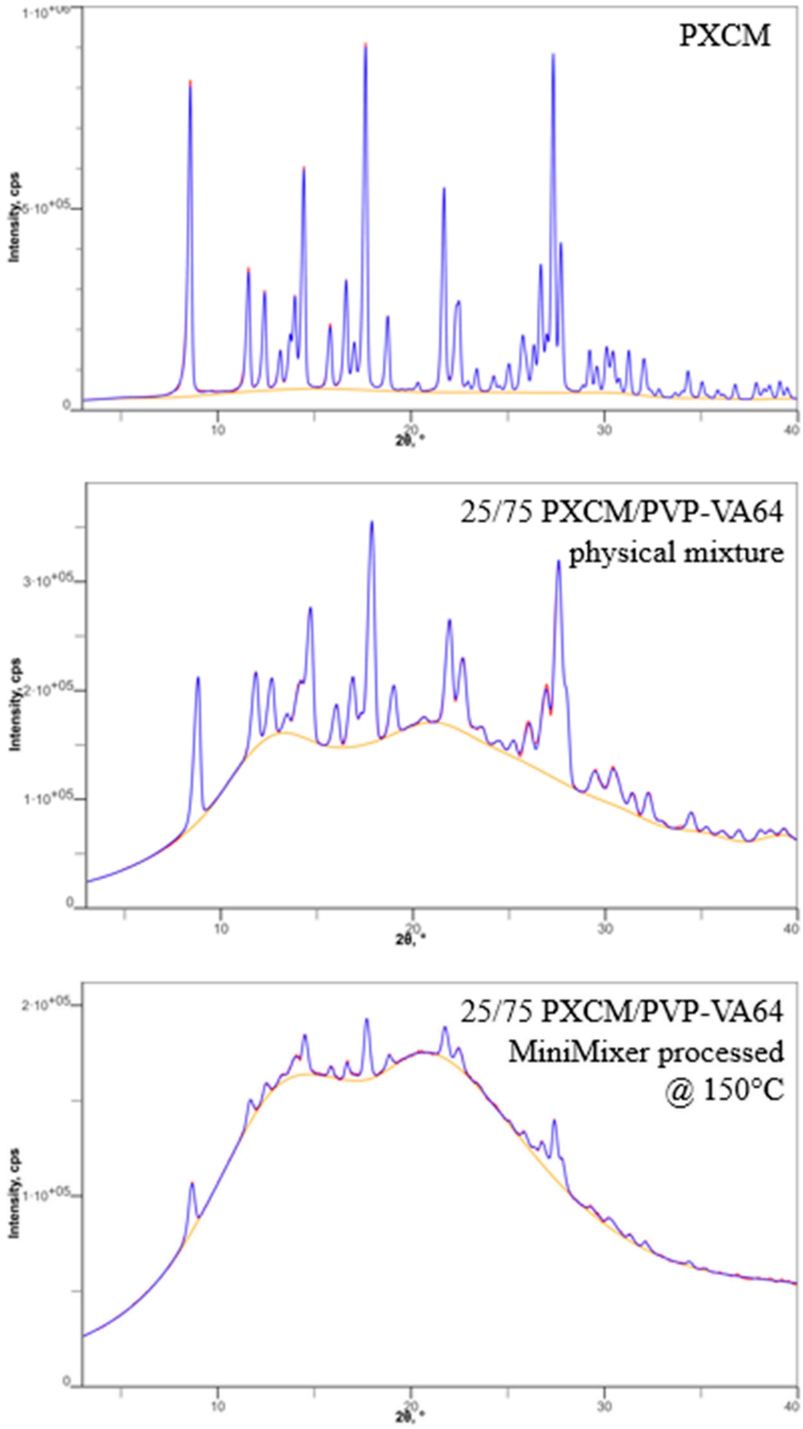

Figure S2. Results of the fitted diffractograms (blue) to the experimental diffractograms (red) against the fitted backgrounds (yellow) for PXCM (top), the 25% physical mixture (middle), and the MiniMixer processed sample (bottom).

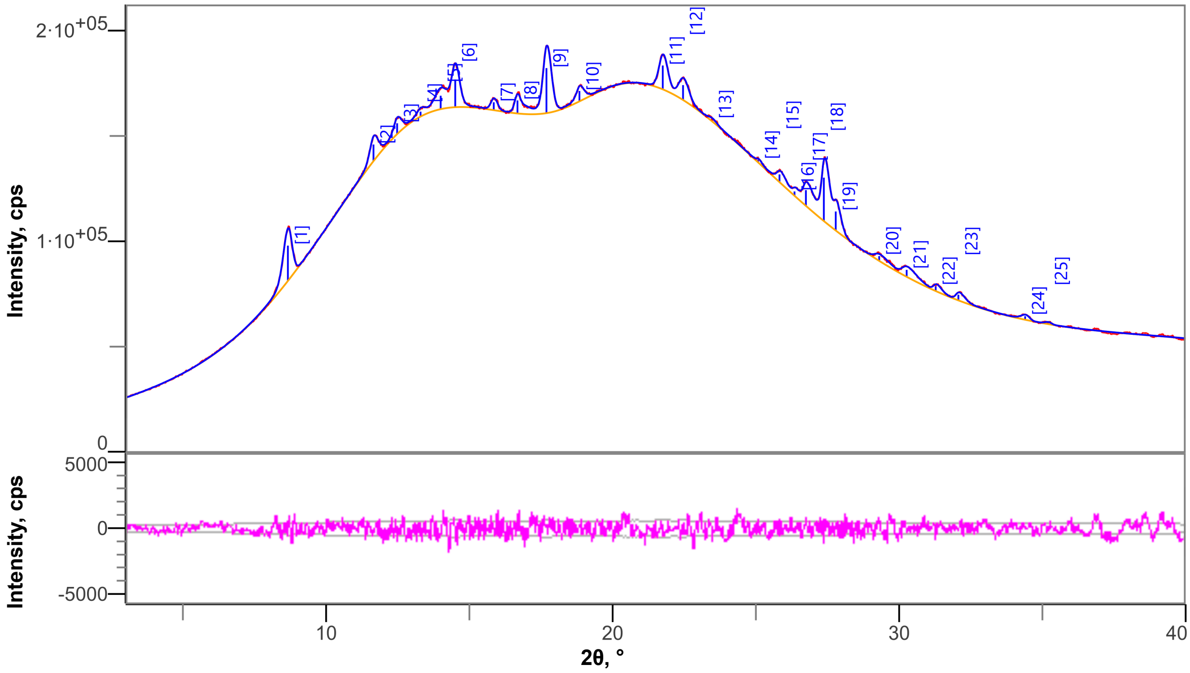

Figure S3. Results of the fitted diffractogram (blue) to the experimental diffractogram (red) against the fitted background (yellow) for the 25% PXCM MiniMixer processed sample, also showing the identified peaks and the residuals trace (pink).

Using the fitted peaks of the processed sample, they are compared to the ingoing physical mixture. Quantitation of solubilized API was determined using the ratio of the peak heights in the samples using the following equations

$$Undissolved\ API\ (\%) = API_{PM} \times \left(\frac{h_{HME}}{h_{PM}}\right)$$

$$Solubilized\ API\ (\%) = API_{PM} - Undissolved\ API$$

$$Acheivable\ API\ loading\ (wt\%) = \frac{Solubilized\ API}{(\text{Solubilized API} + Poly_{PM})} \times 100$$

where API_{PM} is the wt% of the API in the ingoing physical mixture, h_{HME} and h_{PM} are the peaks heights for an API peak at a specific 2θ value in the processed sample and ingoing physical mixture, respectively, and $Poly_{PM}$ is the wt% of the polymer in the ingoing physical mixture. The results for the 2 MiniMixer processed samples are shown in Table S1.

Table S1. Summary of PXCM loading quantitation comparing peak ratios to the ingoing physical mixture

	Ratio of peak heights to the ingoing physical mixture	
2-theta	Sample 1	Sample 2
8.6	0.187	0.149
11.8	0.171	0.141
12.6	0.144	0.124
14.5	0.160	0.121
17.7	0.156	0.125
21.7	0.173	0.147
27.3	0.158	0.145
Average peak ratio	0.164	0.136
% crystalline (undissolved)	4.1	3.4
% amorphous (solubilized)	20.9	21.6
Achievable API loading (wt%)	21.8	22.4
Predicted Loading (wt%)	22.1	

Alternatively, the quantitation can be performed referencing the ingoing API, where the undissolved portion is first calculated by the following equation

$$Undissolved\ API\ (\%) = \left(\frac{h_{HME}}{h_{API}}\right) \times 100$$

where h_{API} is the peak height for an API peak at a specific 2θ value in the ingoing API. Quantifying against the API yields very similar results as the quantitation against the physical mixture, as shown by Table S2. However, using the physical mixture is recommended in order to ensure consistent baseline fitting of the amorphous halo.

Table S2. Summary of PXCM loading quantitation comparing peak ratios to the ingoing API

2-theta	Ratio of peak heights to the ingoing API	
	Sample 1	Sample 2
8.6	0.032	0.033
11.8	0.040	0.043
12.6	0.031	0.036
14.5	0.036	0.035
17.7	0.036	0.038
21.7	0.032	0.034
27.3	0.034	0.039
Average peak ratio	0.034	0.037
% crystalline (undissolved)	3.4	3.7
% amorphous (solubilized)	21.6	21.3
Achievable API loading (wt%)	22.3	22.1
Predicted Loading (wt%)	22.2	

Shear Rate calculations

While the shear in the MiniMixer is within the same order of magnitude as the average shear of an extruder, the maximum shear a material experiences in an extruder is significantly higher than in the MiniMixer. Table S3 details some example calculations of peak shear rates expected for some common extruder configurations, using the overflight gap width (the space between the edge of the screw threads and barrel wall)

$$Shear\ rate\ (s^{-1}) = \frac{\pi * D * n}{h * 60}$$

Table S3. Example calculations of the peak shear rates expected from various extrusion equipment

**Device**	**Screw diameter *D* (mm)**	**Gap width *h* (mm)**	**Screw speed *n* (rpm)**	**Peak shear rate (s^{-1})**
MiniMixer	4 (stirring rod)	0.5	520	218
Micro compounder	14 (back)[a]	0.3[b]	200	489
	5 (front)[a]			175
	14 (back)[a]	0.1[b]	200	1466
	5 (front)[a]			524
Extruder	18	0.3	200	628
			500	1571
		0.1	200	1885
			500	4712
	27	0.3	200	942
			500	2356
		0.1	200	2827
			500	7069

[a] measurements from [I. Berton, R. Castellani, L. Sardo, R. Valette, B. Vergnes, Theoretical and experimental study of the flow of a molten polymer in a micro-compounder, 61 (2021) 3135-3146.]

[b] gap width for the micro compounder used in this study was not provided, so both common gap widths for extruders are calculated for example purposes

PXRD of micro-compounder samples at different temperatures

Figure S4 shows the diffractograms of the samples prepared on the micro compounder at various temperatures. All samples appear amorphous, except the PXCM sample prepared at 130°C. These results are all aligned with expectations from MiniMixer studies.

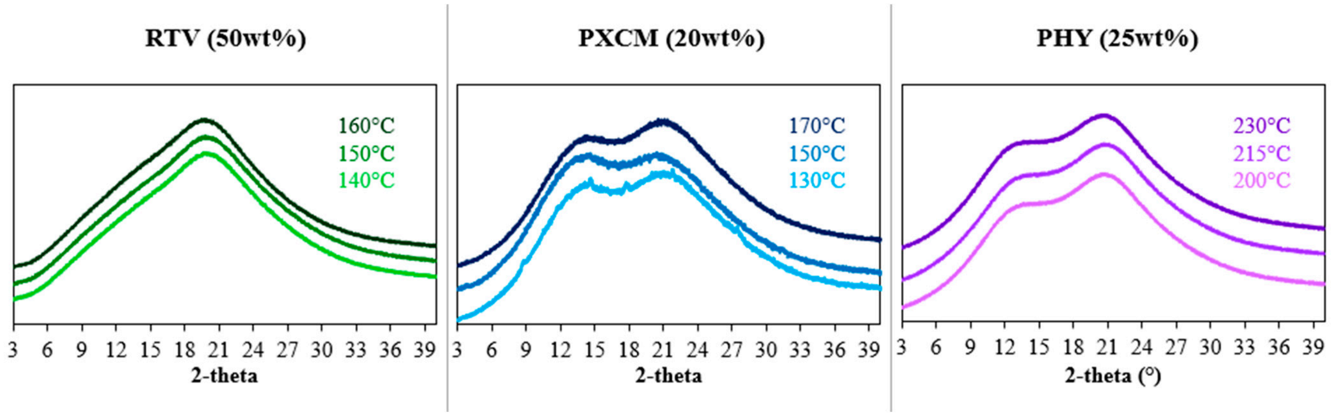

Figure S4. Diffractograms for the RTV, PXCM, and PHY PVP-VA64 samples processed at different temperatures using the micro compounder.

Additional PHY data

Additional MiniMixer tests with PHY to demonstrate the reliability of the MiniMixer at various conditions and with other polymers was conducted. A 2nd temperature, 170°C, was also tested for the PHY/PVP-VA64 system, and HPMCAS-M was tested as another polymer. Following the same procedures described in the paper, mixtures with varying PHY loadings were prepared, based on the achievable API loading predictions from MiniMixer studies, and processed on the micro compounder for 3 minutes. The results are shown in Figure S5. Significantly higher loadings are possible in PVP-VA64 (between 20 and 25wt%) compared to HPMCAS-M (less than 15wt%), but good MiniMixer-to-micro compounder correlations are demonstrated in all cases.

It should be noted that the PHY lot used for these studies is different than the one used for all work described in the main text. The lot used to generate the data in Figure S5 is of a lower starting purity, and contains what is believed to be NaCl impurity, as evidenced by the peak at ~31 2θ.

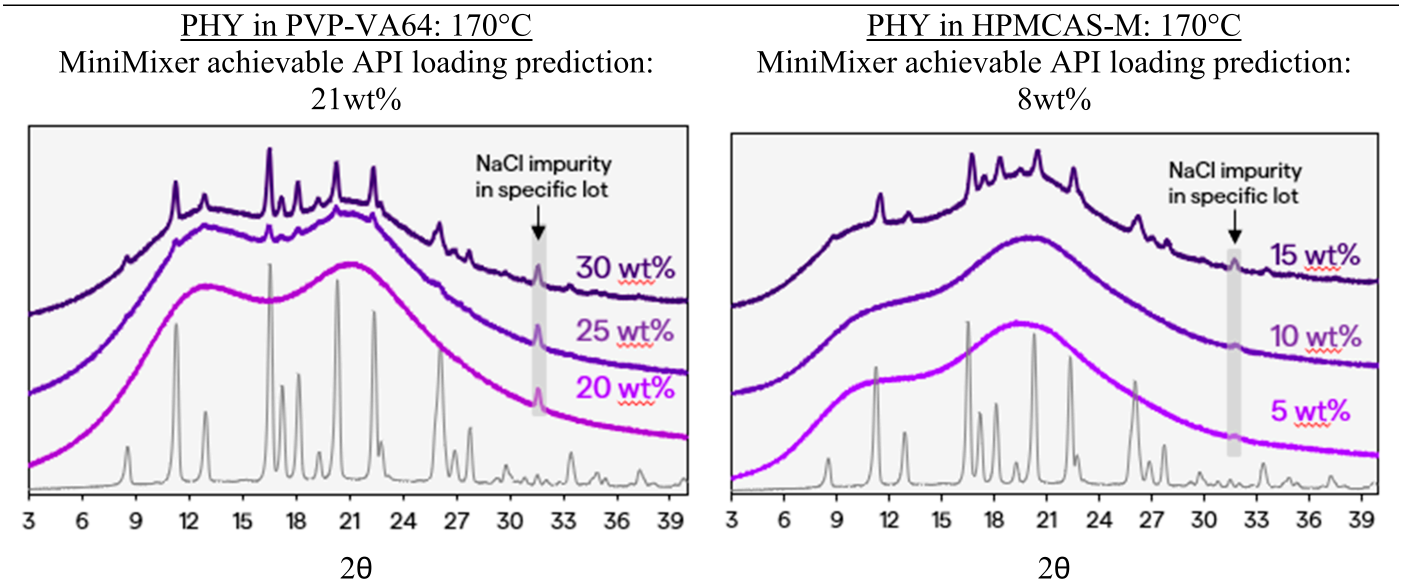

Figure S5. Diffractograms of PHY samples of various API loadings prepared on the micro compounder at 170°C and 3 minutes of mixing with two different polymers, PVP-VA64 (left) and HPMCAS-M (right)

Visual Observations of processed samples

Images of the processed samples are shown in Figure S6. RTV shows the least amount of visual change of all the samples, with only very subtle browning observed beyond the 1 hour timepoint for the unmixed isothermal holds. PHY also shows subtle changes across samples, but slight discoloration is observed in the micro compounder samples as well as the longer times of the unmixed isothermal holds. The color change was more obvious before milling, where it was noted that the 2 and 4 hour isothermal holds appeared bright orange in color. PXCM shows significant yellowing and browning in all samples. Even the micro compounder sample processed at 130°C appears bright highlighter yellow.

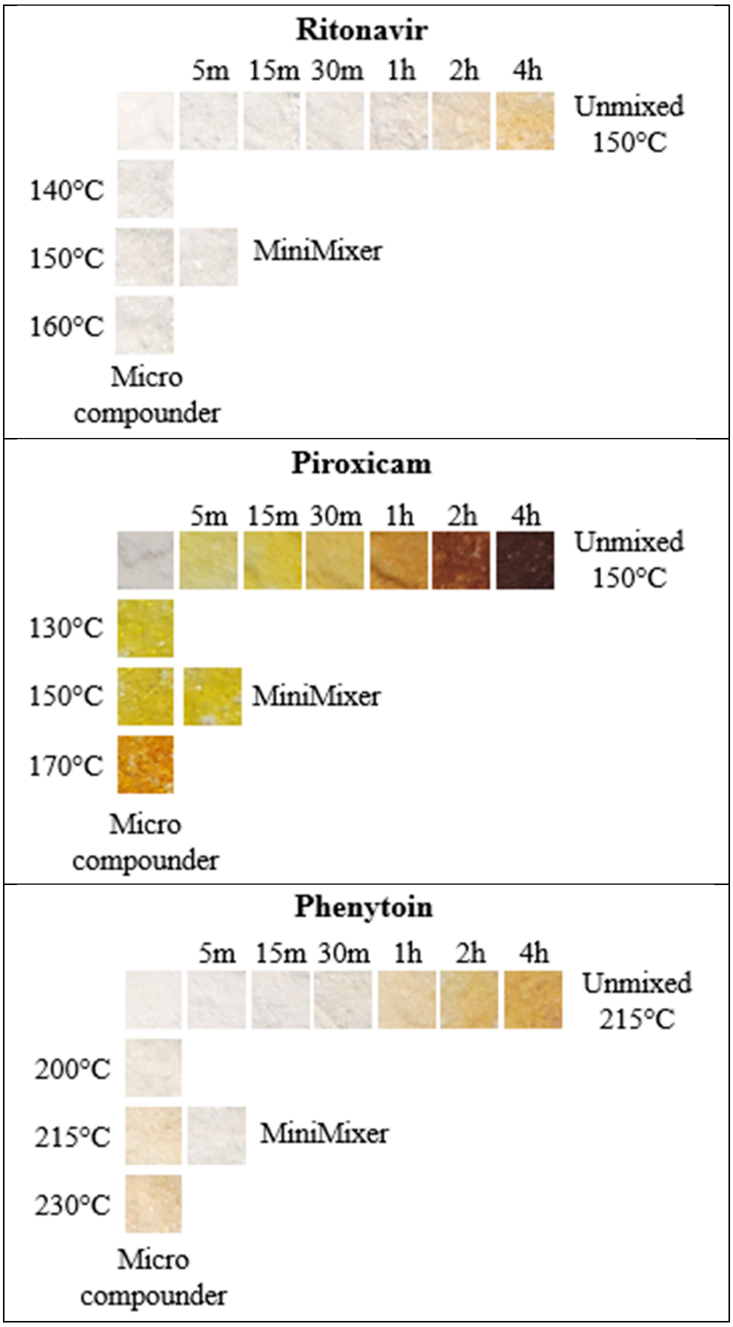

Figure S6. Images of the samples processed under various conditions. For all three systems, the top left square shows the ingoing physical mixture, and the top row shows the unmixed isothermal holds over time. The first column shows the micro compounder samples prepared at 3 different temperatures, and the MiniMixer processed sample at the middle temperature is shown to the right.

Thermal Analysis by DSC

DSC was run to determine the glass transition temperature of the MiniMixer and micro compounder processed samples, to get a sense of solubilized API and determine whether the materials from both processes were physically similar or not. The individual transition temperatures of the neat APIs were not measured, but expected to be less than the PVP-VA64 T_g of 108°C. The resultant ASD should therefore have a T_g lower than the pure polymer, decreasing with increased amorphous (solubilized) API content. All samples showed a single T_g as summarized in Table S4.

Table S4. Summary of glass transition temperatures as determined by DSC for MiniMixer and micro compounder processed samples.

API	Processing T (°C)	Loading (wt%)	Processing Method	Onset (°C)	T_g (°C)	End (°C)	Span (°C)	ΔCp (J/g°C)
		20	Micro compounder	87.1	**93.0**	98.9	11.8	0.23
**PXCM**	150	25		83.6	**89.8**	96.0	12.5	0.20
		25	MiniMixer	86.9	**93.0**	99.2	12.3	0.26
**RTV**	150	50	Micro compounder	59.7	**62.2**	64.8	5.1	0.31
		50	MiniMixer	60.1	**62.3**	64.5	4.4	0.23
		10		102.1	**107.1**	112.2	10.1	0.38
		25	Micro compounder	100.8	**106.0**	111.2	10.4	0.37
**PHY**	215	30		100.5	**105.7**	110.9	10.3	0.39
		35		100.0	**105.2**	110.4	10.4	0.32
		35	MiniMixer	100.7	**106.0**	111.3	10.6	0.34

Both of the RTV samples, amorphous by PXRD, result in the same T_g of 62°C indicating they are physically similar materials with all API dispersed into the polymer.

For PXCM, the MiniMixer sample with 25wt% API shows the same T_g as the 20wt% micro compounder processed sample. Quantitation shows the 25wt% MiniMixer processed sample to have ~20-22wt% solubilized API (see Table S1 and STable S2), while the 25wt% micro compounder processed sample is near fully amorphous. Overall, the DSC data lines up with the XRD quantitation results.

Due to the similarities in T_g between the 25 and 30wt% PHY micro compounder samples (both amorphous by PXRD), a 10wt% extrudate was also prepared to better understand the T_g v. API loading profile, as plotted in Figure S7. The impact of the API to the T_g of the system is very subtle: with error bars, the 10wt% extrudate overlaps with both the pure polymer and 25wt% extrudate. Making API loading predictions from the DSC data would therefore be difficult to decipher. Additionally, the unmixed samples exhibit a Tg closer to the 10wt% extrudate despite the PXRD data suggesting nearly a 30wt% amorphous sample, and shows an increase over time (Figure S9). This is believed to be caused, at least in part, by polymer degradation: pure PVP-VA64 held at 215°C for 4 hours resulted in a 3°C increase of the T_g as shown in Table S5. This could also be in part due to inhomogeneity in the system, with the API not fully dispersed into the polymer molecularly, but phase separation would not be detectable due to T_g similarities.

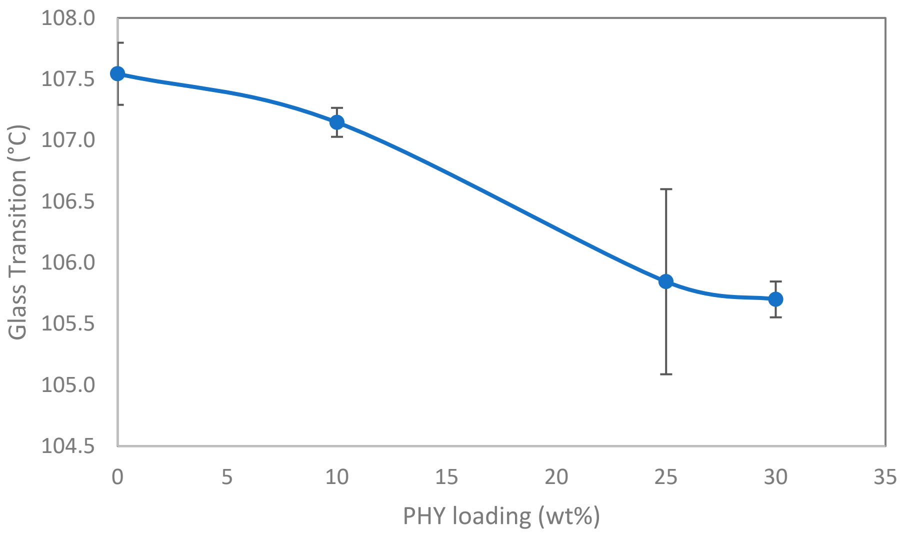

Figure S7. Glass transition temperature versus API loading for the PXRD-amorphous PHY samples made on the micro compounder along with the bulk PVP-VA64.

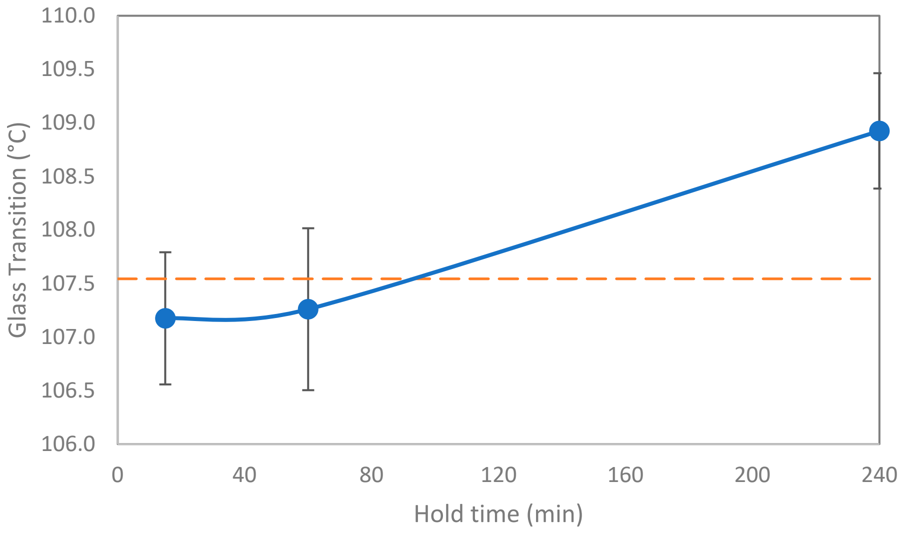

Figure S8. Glass transition temperature versus time at temperature for the PHY unmixed samples held at 215°C. The dashed line shows the Tg of the unprocessed PVP-VA64

Table S5. Glass transition temperature of PVP-VA64 before and after a 4 hour isothermal hold at 215°C

Sample	Hold T (°C)	Hold time (h)	Onset (°C)	T_g (°C)	End (°C)	Span (°C)	ΔCp (J/g°C)
PVP-VA64	215	0	102.4	**107.5 ± 0.3**	112.7	10.3	0.38
		4	105.7	**110.8 ± 0.2**	116.0	10.3	0.40

Approximately 3-5mg of sample was placed into a Tzero standard DSC pan and gently tapped to uniformly disperse. A lid was placed on the sample and secured into place using the Tzero press. Samples were analyzed with either a TA Q2000 C or a TA Discovery DSC2500, scanned from 0 to 150°C at 2.5C/min with ± 1.5°C/min modulation. Glass transition temperatures were analyzed on the reversing signal with midpoint determination. Samples were run in triplicate.

MiniMixer Temperature Monitoring

Extruders generally need to be fitted with cooling jackets to help control the barrel temperatures, as excess heat due to viscous dissipation builds up. Temperature monitoring of a PVP-VA64 placebo inside of the MiniMixer was conducted to determine whether this would be a concern. The results, summarized in Figure S9, indicate that the sample temperature may increase by 5-10°C during mixing, but does not increase with mixing duration.

The steady decrease in temperature seen when mixing is stopped shows that the act of mixing does cause the temperature to increase. There does not appear to be a significant rise in temperature once the mixing has begun though, so concern that longer mixing times result in higher temperatures is invalid. The noise observed during active mixing is mainly due to the physical contact of the thermocouple probe with the stirring rod. Because of the viscous polymer, it is difficult to hold the thermocouple in place without making contact with the stirring rod and/or causing it to get slightly wrapped.

Another observation from Figure S9 is that the sample temperature is below the set temperature. This could mean that solubility results from the MiniMixer are actually related to a lower temperature than reported. However, it should be noted that a small portion of the thermocouple probe does not sit inside the cavity, and is therefore exposed to lower temperatures. It is likely that this contributes to the experimental temperatures being lower than the set temperature of the heating block.

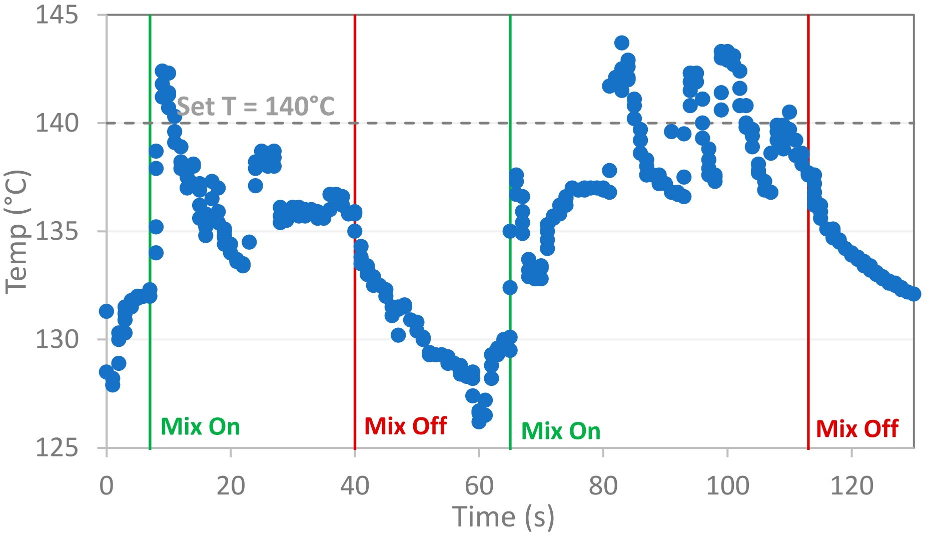

Figure S9. Temperature monitoring of PVP-VA64 in the MiniMixer with and without mixing applied

To conduct this experiment a Digi-Sense type K thermocouple probe 0.75 inches long and 0.02 inches in diameter (Cole-Parmer PN: 08505-96) was placed inside the cavity of the MiniMixer after adding the polymer and inserting the stirring rod. To monitor the temperature with time, a timer was placed by the Fluke digital temperature readout and filmed for the duration of the experiment. Temperature as a function of time was transcribed afterwards.
